# Supplementary material for: Do Patients with Autoimmune Conditions Have Less Access to Liver Transplantation despite Superior Outcomes?
Source: J Pers Med. 2022 Jul 17;12(7):1159. doi: 10.3390/jpm12071159 (PMC9320508; doi:10.3390/jpm12071159)
Supplement: Supplementary file 1 [file jpm-12-01159-s001.zip › jpm-1795157-supplementary.pdf]

Table S1. Multivariate Probability of Transplant for All Ages and Under 40.

Cox Regression Analysis: Multivariate Probability of Transplant						
**Listing Diagnosis**	**Probability of Transplant HR(95% CI)**					
	2000-2004	2005-2009	2010-2014	2015-2019	2020- July 2021	All Eras
Autoimmune Conditions	0.92 (0.91 , 0.93)	0.92 (0.91 , 0.93)	0.91 (0.90 , 0.92)	0.93 (0.92 , 0.94)	0.93 (0.90 , 0.95)	0.92 (0.92 , 0.93)
Alcohol Related Disease	0.97 (0.96 , 0.98)	0.98 (0.97 , 0.98)	0.96 (0.95 , 0.97)	0.96 (0.95 , 0.97)	0.97 (0.95 , 0.98)	0.97 (0.97 , 0.97)
Hepatocellular Carcinoma	1.13 (1.11 , 1.14)	1.12 (1.11 , 1.13)	1.09 (1.08 , 1.10)	1.03 (1.02 , 1.04)	0.96 (0.94 , 0.97)	1.08 (1.07 , 1.08)
Viral Hepatitis	0.99 (0.98 , 0.99)	0.99 (0.99 , 1.00)	1.00 (0.99 , 1.00)	1.00 (0.99 , 1.00)	0.97 (0.96 , 0.98)	0.99 (0.99 , 0.99)
NASH	1.00 (0.98 , 1.03)	0.98 (0.97 , 0.99)	0.98 (0.97 , 0.99)	0.98 (0.97 , 0.99)	0.98 (0.97 , 0.99)	0.99 (0.99 , 1.00)

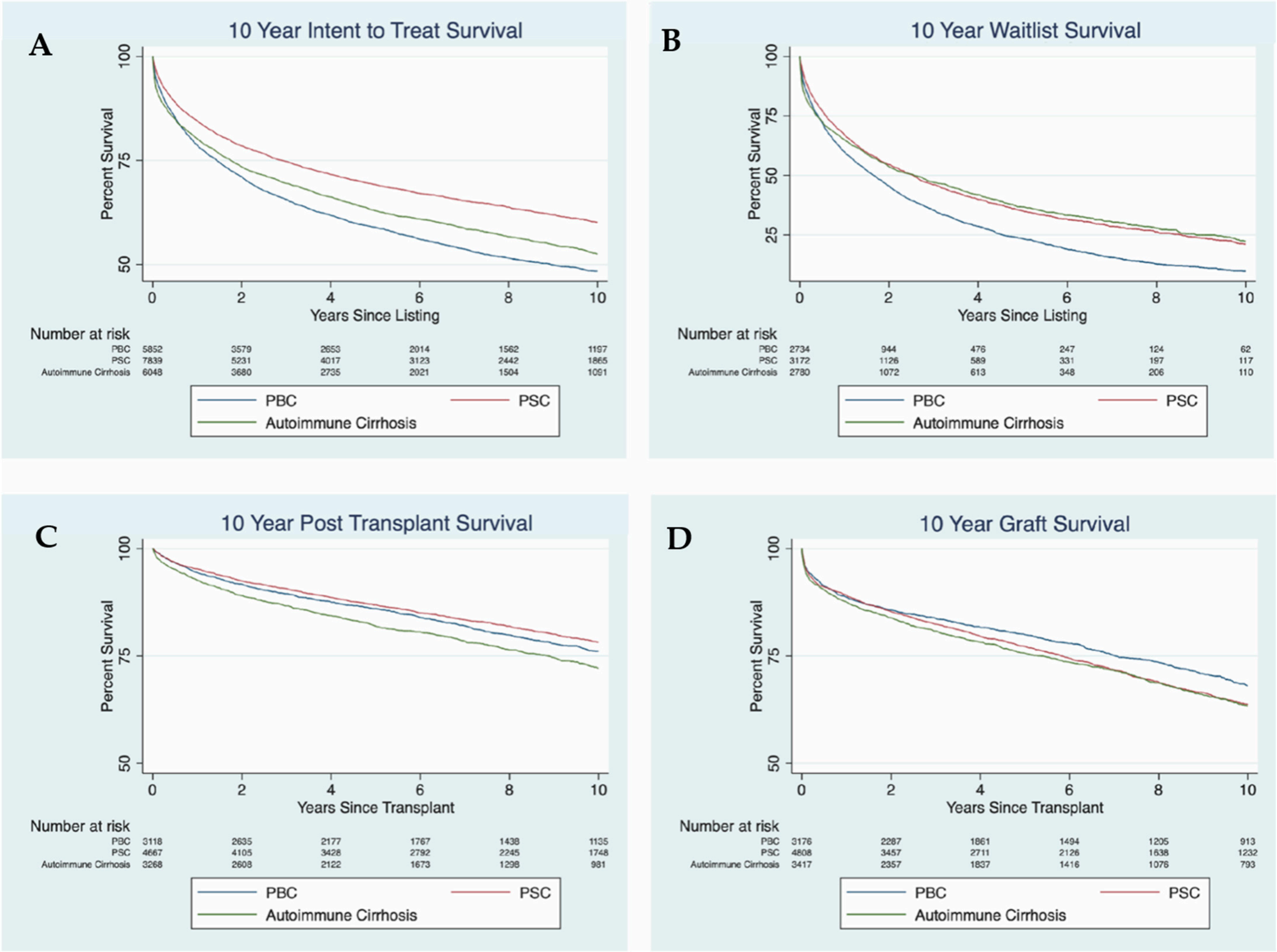

Figure S1. Top Left: ITT Survival, Top Right: Waitlist Survival, Bottom Left: Post-Transplant Survival, and Bottom Right: Graft Survival for Individual Autoimmune Conditions. (A) ITT Survival for adults listed with PBC, PSC, and Autoimmune Cirrhosis. All curves are significantly different from other curves (p-value < 0.001, log rank test) (B) Waitlist Survival for adults listed with PBC,

PSC, and Autoimmune Cirrhosis. PBC significantly different from other curves (p-value < 0.001, log rank test). PSC and Autoimmune Cirrhosis not significantly different from each other (p-value = 0.4074, log rank test) (C) Post-transplant survival for adults transplanted with PBC, PSC, and Autoimmune Cirrhosis in all eras for all adults. All curves are significantly different from other curve (p-value < 0.001, log rank test). (D) Graft survival for adults transplanted with PBC, PSC, and Autoimmune Cirrhosis in all eras for all adults. PBC vs. PSC and PBC vs. Autoimmune Cirrhosis significantly different (p-value = 0.0038, p-value < 0.001 respectively, log-rank test), PSC vs. Autoimmune Cirrhosis curves are not different from each other (p = 0.237, log rank test).
